# *Pinelliae rhizoma* alleviated acute lung injury induced by lipopolysaccharide *via* suppressing endoplasmic reticulum stress-mediated NLRP3 inflammasome

**DOI:** 10.3389/fphar.2022.883865

**Published:** 2022-08-15

**Authors:** Ning-ning Wang, Xian-xie Zhang, Pan Shen, Cong-shu Huang, Hui-fang Deng, Lei Zhou, Lan-xin Yue, Bao-ying Shen, Wei Zhou, Yue Gao

**Affiliations:** ^1^ Tianjin University of Traditional Chinese Medicine, Tianjin, China; ^2^ Department of Pharmaceutical Sciences, Beijing Institute of Radiation Medicine, Beijing, China; ^3^ Guangdong Pharmaceutical University, Guangzhou, China

**Keywords:** *Pinelliae rhizoma*, acute lung injury, NLRP3 inflammasome, metabolomics, endoplasmic reticulum stress

## Abstract

*Pinelliae rhizoma* (PR), one kind of commonly-used Chinese herbs, is generally prescribed to treat various respiratory diseases, including acute lung injury (ALI). However, the accurate bioactive ingredients of PR and the underlying pharmacological mechanism have both not been fully elucidated. Therefore, this study aimed to identify the bioactive ingredients that could alleviate lipopolysaccharide (LPS)-induced ALI and explore the possible mechanism involved. Our results confirmed that LPS infection indeed caused acute inflammatory damage in mice lung, accompanying with the enhancement of IL-1β contents and the activation of the NLRP3 inflammasome in lung tissue and macrophagocyte, all of which were remarkably ameliorated by PR treatment. Next, mechanistically, LPS was found to trigger endoplasmic reticulum (ER) stress and downstream cellular calcium ions (Ca^2+^) release *via* activating Bip/ATF4/CHOP signaling pathway. Like PR, 4-PBA (a specific inhibitor of ER stress) not only obviously reversed Bip/ATF4/CHOP-mediated ER stress, but also significantly attenuated LPS-induced activation of the NLRP3 inflammasome. Furthermore, the bioactive ingredients of PR, which generated the anti-inflammatory effects, were screened by metabolomics and network pharmacology. *In vitro* experiments showed that chrysin, dihydrocapsaicin, and 7,8-dihydroxyflavone (7,8-DHF) notably suppressed LPS-induced ER stress and following NLRP3 inflammasome activation. In conclusion, our findings suggested that PR alleviated LPS-induced ALI by inhibiting ER stress-mediated NLRP3 inflammasome activation, which is mainly relevant with these three bioactive ingredients. This study provided a theoretical basis for the clinical application of PR to treat ALI, and these bioactive ingredients of PR would be promising therapeutic drugs for the treatment of ALI.

## Introduction

The global pandemic of severe acute respiratory syndrome coronavirus 2 (SARS-CoV-2) causes numerous severe pneumonia (named coronavirus disease 2019, COVID-19) and death, both of which are associated with SARS-CoV-2 induced acute lung injury (ALI) or its wilder form, acute respiratory distress syndrome (ARDS) (Habashi et al., 2021). Several Traditional Chinese Medicine (TCM) formulas have been reported to perform well against progressing to severe or critical COVID-19, largely because these prescriptions generated immune regulatory effects to prevent ALI or further ARDS (Huang et al., 2021). Of these TCM formulas used for the treatment of COVID-19, five kinds of herbs were documented as clinical commonly-used Chinese medicinal materials to treat and prevent various pulmonary diseases, including *Glycyrrhiza uralensis Fisch* ex DC. *[Fabaceae], Scutellaria baicalensis Georgi [Lamiaceae]*, *Pinellia ternata (Thunb.) Makino [Araceae]*, *Forsythia suspensa (Thunb.) Vahl [Oleaceae]*, and *Semen Armeniacae Amarum [Rosaceae]* (Xiong et al., 2020; Luo et al., 2022). In particular, for its excellent efficacy in the therapy of chronic obstructive pulmonary disease (COPD), asthma, and respiratory tract infections, *Pinelliae rhizoma* (PR) has been widely used in many East Asian countries (Du et al., 2016; Hu et al., 2019). Growing studies are paying attention to the material basis and mechanism involved in the treatment of ALI using PR.

In general, ALI is considered to be an adverse outcome of immunological responses to bacterial or viral agents’ infection, in addition to the key event in deciding deterioration of diseases (Mokra, 2020). Next, pathologically, ALI is characterized by elevated alveolar permeability and protein-rich edema, both of which are always caused by hyper-activated pro-inflammatory responses and following tissue damage (Kumar, 2020). Hence, it's not difficult to spot that controlling inflammatory injury would be the crux of ALI therapy. Previous studies found that PR exerts protective effects *via* attenuating allergic airway inflammation and mucus excessive secretion in asthma murine models and also relieves chronic airway inflammation in rats with COPD (Lee et al., 2013; Lyu et al., 2020). Moreover, Tang X et al. reported that the combination of PR or its bioactive ingredient (β-sitosterol) with another herb indeed decreased the contents of inflammatory factors and ameliorated lung injury in lipopolysaccharide (LPS)-infected rat (Tang et al., 2018). However, the molecular mechanisms underlying PR-generated anti-inflammatory effects during the therapy of ALI are not fully elucidated. Besides, accumulating evidence demonstrated that PR contains plenty of bioactive ingredients, for example, alkaloids, anthraquinone glycosides, and their derivatives (Mao and He, 2020). Therefore, an urgent need also exists to identify the bioactive ingredients of PR that are associated with ALI treatment.

NF-κB signaling and the downstream nucleotide-binding oligomerization domain, leucine-rich repeat, and pyrin domain-containing 3 (NLRP3) inflammasome act as the core modulators that mediate the outbursts of pro-inflammatory cytokines, including IL-1β, TNF-α, and IL-18, and subsequent cell death during the over-activated inflammatory process of ALI (He et al., 2016). In recent times, endoplasmic reticulum (ER) stress, a cellular response to ensure proteins fold correctly, was initiated upon sensing LPS or virus infection to participate in the occurrence and development of ALI by regulating immunological recognition, macrophage activation, and alveolar endothelial function (Hrincius et al., 2015; Zhang et al., 2018; Zhao et al., 2020). Next, typically, the activation of ER stress triggers unfolded protein response (UPR) to restore cellular homeostasis (Kim et al., 2015) and programmed apoptosis *via* C/EBP homologous protein (CHOP) once protein misfolding occurs beyond repair capacity (Delbrel et al., 2018). Besides, over-activation of ER stress causes the cytoplasmic release of calcium ions from ER, resulting in the activation of the NLRP3 inflammasome and mitochondrial dependent death (Li et al., 2020; Mishra et al., 2021). Latest studies also showed that the cross-talk between ER stress and IKKbeta/NF-κB signaling cascade was relevant to many pathological events, for instance, energy imbalance, autophagy, and apoptosis (Zhu et al., 2017; Lee et al., 2021). Thus, the interplay among ER stress and these pro-inflammatory signals would promote and exacerbate ALI. However, whether ER stress is targeted by bioactive ingredients of PR during ALI treatment remains unknown.

**FIGURE 1:**
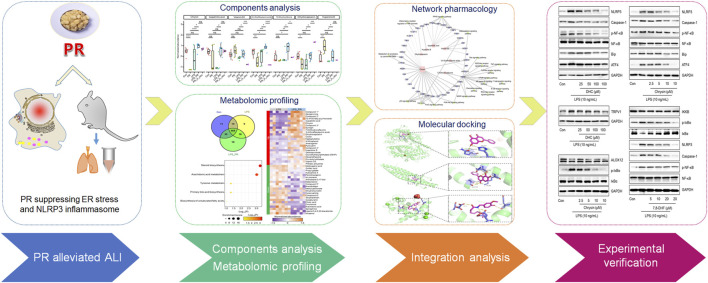
Flowchart of the research process. PR alleviates the damage caused by LPS, and its active ingredients were explored by metabolomics and validated *in vitro*.

Here, we speculate that PR may alleviate lung injury *via* inhibiting ER stress-mediated pro-inflammatory responses. In this study, we confirmed that PR treatment alleviated LPS-induced ALI *in vivo* and *in vitro*, and we also identified the relevant bioactive ingredients by metabolomics. Next, mechanistically, PR and these bioactive ingredients provided relief from acute inflammatory injuries mainly by inhibiting ER stress-mediated NLRP3 inflammasome, which could be a novel therapeutic target for ALI. The flow chart of the research processis shown in Figure 1.

## Materials and methods

### Chemicals and reagents

LPS (L8880, HPLC ≥ 99%, China), 7,8-dihydroxyflavone (7,8-DHF, CAS: 38183038, HPLC ≥ 98%), and dihydrocapsaicin (DHC, CAS:19408845, HPLC ≥ 98%) were obtained from Beijing Solarbio Science & Technology Co. Ltd. Chrysin (CAS: 480400, HPLC ≥ 98%), 2-aminoethoxydiphenyl borate (2-APB, #9754, HPLC ≥ 97%), Phosphate Buffered Saline, and formic acid (LC-MS grade) were all purchased from Sigma-Aldrich (Merck, United States). Next, Sodium 4-phenylbutyrate (4-PBA, T1535, HPLC ≥ 99%) was purchased from Topscience Co. Ltd. Sodium chloride injection was obtained from Shijiazhuang NO.4 Pharmaceutical Co. Ltd., (Shijiazhuang, China). Methanol (LC-MS grade) and acetonitrile (LC-MS grade) were obtained from CNW Technologies (Shanghai, China). 2-Chloro-L-phenylalanine (≥ 98%, HPLC) was purchased from Hengbai Co, Ltd. (Shanghai, China), and other reagents were HPLC grade.

Anti-IL-1β (511369) and anti-Caspase-1 (342947) were purchased from Zen-bioscience Biotechnology Co. Ltd. (Chengdu, China). Anti-NLRP3 (ab263899) was obtained from Abcam (Cambridge, MA, United States). Anti-ASC/TMS1 (67824), anti-Bip (3177), anti-ATF4 (11815), anti-IKKβ (8943), anti-phospho-IκBα (14D4), anti-IκBα (L35A5), anti-phospho-NF-κB p65 (93H1), anti- NF-κB p65 (8242), and anti-GAPDH (5174) were all obtained from Cell Signaling Technology (Beverly, MA, United States). Anti- ALOX12 (A02275) was purchased from Boster Biological Technology Co. Ltd. (Wuhan, China). Anti-CHOP (15204) and anti-TRPV1 (66983) were obtained from Proteintech (Wuhan, China).

### Preparation of the *Pinelliae rhizoma* aqueous extract

PR used in this study was *Pinelliae rhizoma Praeparatum Cum Alumine*, the processed product of the dried rhizome of *Pinellia ternata (Thunb.) Makino [Araceae]*, and it was purchased from Minghui-Hengtong Pharmaceutical Co. Ltd. (LOT NO.: 20030102, Beijing, China). Professor Yan Jin, National Resource Center for Chinese Materia Medica authenticated the PR according to the Pharmacopeia of the People’s Republic of China (2020, volume I). The extraction procedures of PR used for *in vivo* experiments are as follows: PR (50 g) was immersed in distilled water (1 L) for 30 min, followed by boiling and decocting twice for 30 min each time. All decoctions were combined, and the extracts were filtered through a Buchner funnel. For *in vivo* experiments, the filtrates were concentrated under reduced pressure to 500 mg/ml at 60–70°C and stored at −20°C. For *in vitro* experiments, the filtrates were freeze-dried to obtain a dry fluffy powder (the drug extract ratio based on the mass was 6.5 g: 50 g) and stored at −20°C. In addition, we also carried out quality control of PR extract samples and listed the peaks of major ingredients of PR*.* Researchers identified by mass spectrometry that the water extract of PR contains succinate, adenine, ferulic acid, baicalein, and coumarin. For detailed data, please refer to Supplementary Data Sheet S1 and Supplementary Table S3.

### Instruments

UHPLC system (Vanquish, Thermo Fisher Scientific, United States); Chromatographic column (ACQUITY UPLC BEH C18 (1.7 μm 2.1*100 mm), Waters, United States); High resolution mass spectrum (Orbitrap Exploris 120, Thermo Fisher Scientific, United States); Analytical Balance (Mettler Toledo, Swiss); High-throughput tissue grinder (Shanghai, China); Pure water filter (Merck Millipore, United States); Ultrasonic cleaner (Fangao Co., Ltd., Shenzhen, China); CO_2_ incubator (ThermoFisher, United States); Multifunctional enzyme marker (PerkinElmer, United States); Ultra low temperature refrigerator (Haier, China).

### Cell culture and treatments

RAW264.7 cells were obtained from the National Infrastructure of Cell Line Resource. Cells were grown in DMEM medium (Gibco, ThermoFisher Scientific, United States) containing FBS (10%) and penicillin-streptomycin (1%). Next, RAW264.7 cells were cultured in a cell incubator at 37°C with 5% CO_2_. When the cell density reached 80%, drug treatment was performed.

### Animals and experimental design

C57BL/6 mice (8–10 weeks) were ordered from Beijing Vital River Laboratory Animal Technology (Beijing, China). Mice were kept in standard laboratory with unlimited access to standard diet and water. They were randomly divided into five groups (*n* = 6): control group, LPS-stimulated model group, PR-low dosage groups (1 g/kg/day), PR-medium dosage groups (2 g/kg/day), and PR-high dosage groups (4 g/kg/day). The Pharmacopeia of the People’s Republic of China (2020, volume I) stipulates that the maximum dose of PR is 9 g per person per day. According to the dose conversion guideline between mice and human stipulated by the FDA (Reagan-Shaw et al., 2008), the dose of PR administered in mice was 2 g/kg/d, so the low dose (1 g/kg/d) and high dose (4 g/kg/d) were 0.5 times and 2 times that of the middle dose group, respectively. The applied dose of this study demonstrates guiding significance for clinical medication. The mice model of ALI was established by intranasal inhalation of LPS at 5 mg/kg. Also, daily changes in weight and clinical signs were recorded during administration. Mice were killed by euthanasia at the end of administration, and the samples were saved for subsequent studies.

### Hematoxylin-eosin staining of lung tissue

Mice were sacrificed after treatment, and lungs were collected and fixed in 10% neutral buffered formalin. Next, the fixed lungs were embedded in paraffin and cut into sections, followed by H&E staining to detect pathological damage in mouse lung tissue.

### Western blot analysis

Group assignment and drug administration were performed according to the above method. Lung tissue and RAW264.7 cells were homogenized ice-cold RIPA lysis buffer. Total proteins for each group were separated on PAGE gel by electrophoresis and then transferred onto PVDF membrane (Millipore, United States) using the Bio-Rad protein assay system (Bio-Rad, United States). The PVDF membranes were blocked with 5% BSA in TBST buffer for 3 h and then incubated with the corresponding primary antibody overnight at 4°C. The next day, PVDF membranes were washed with TBST and incubated with secondary antibody for 1 h. After thorough washing with TBST, the immunoreactive protein was visualized with an enhanced chemiluminescence assay and captured on ImageQuant^™^ LAS 500 (Healthcare BioSciences AB, United States). Data were standardized with the corresponding GAPDH. All experiments were repeated in triplicate.

### Measurement of cytokine

Mice in each group were euthanized after 7 days treatment of PR, and their serum were harvested from the abdominal aorta using a syringe. The content of cytokine was measured using ELISA kits (IL-1β, MM-0040M1). The absorbency was examined at 540 nm. Next, each value was calculated and presented by deducting the background value.

### Detection of the level of cytoplasmic calcium (Ca^2+^)

Fluo 4-AM (F312, Dojindo, Japan), a Ca^2+^ -specific vital dye, was used to measure intracellular calcium levels. Dilute 1 mM Fluo 4-AM stock solution to 5 μM Fluo 4-AM working solution using HBSS buffer. The working solution (Fluo 4-AM) was incubated in cell incubator for 0.5 h. After washing the RAW264.7 cells three times with HBSS buffer, 1 ml of HBSS buffer was added to continue incubation for 30 min in a cell incubator. After the end, the content of calcium ions in each sample was detected according to the fluorescence intensity using a laser confocal microscope.

### Metabolites extraction

Mice in each group were euthanized after 7 days treatment of PR, and their serum were harvested from the abdominal aorta using a syringe. The blood of the mice in the Con, LPS, and LPS_PR (2 g/kg/d) were centrifuged at 12,000 rpm for 15 min at 4°C. The serum was transferred to another new centrifuge tube, and the extract containing the internal standard solution was added. The samples were centrifuged at 4°C for 15 min, and the supernatant was filtered through a 0.22 μm microporous membrane. 100 μl from each sample was taken and mixed into QC samples. Next, hydrochloric acid (2 mol/L) was added to the serum sample, and it was allowed to stand at 4°C for 15 min. After repeating this for four times, acetonitrile was added. After centrifugation, the supernatant was aspirated to dry with nitrogen. Dried samples were dissolved in 80% methanol, vortexed and centrifuged, and the supernatants were taken for LC/MS detection.

### LC-MS/MS conditions

LC-MS/MS analysis was carried out an UHPLC system coupled with a Waters UPLC BEH C18 column. During the analysis, the injection speed and volume were 0.4 ml/min and 5 μl, respectively. The mobile phase was a combination of 0.1% formic acid in water (A) and 0.1% formic acid in acetonitrile (B). After linear elution, MS and MS/MS data were harvested in IDA acquisition form using an Orbitrap Exploris 120 mass spectrometer.

### Metabolome data processing and analysis

The raw data were processed by Progenesis QI. The area-under-the-curve of each peak was quantified as peak intensity. Peaks with missing values in more than ¼ of the samples were removed. For filtered peaks, missing values were replaced by 1/5 of the min positive value for each variable. To improve the power of subsequent results (Hackstadt and Hess, 2009), further peak filtering was based on interquartile range by R package MetaboAnalystR (version 3.2.0) (Pang et al., 2020). To conclude, the quantile normalized, and log transformed peak intensities were scaling by mean centering. Metabolites were identified by automated comparison using CAMERA (Kuhl et al., 2012). Next, partial least squares-discriminant analysis (PLS-DA) was applied for visualizing group separation and finding significantly changed metabolites by MetaboAnalystR. Welch’s *t*-test was performed for comparing the expression differences of metabolites between each two groups. Metabolites with *p < 0.05* and variable importance in projection (VIP, a weighted sum of squares of the PLS loadings taking into account the amount of explained Y-variation in each dimension) in component 1 > 1.0 were considered as potential biomarkers (Pang et al., 2020).

### Network pharmacology analysis

The targets of PR were forecasted by the webtool SwissTargetPrediction (Daina et al., 2019) with probability > 0.5 in *Homo sapiens*. Also, the ALI-related targets in *Homo sapiens* were obtained from GeneCards (Safran et al., 2010) and OMIM (online mendelian inheritance in man) (Amberger et al., 2015; Amberger et al., 2019). The intersection between the two target sets were the final PR targets for ALI. The PR-target-pathway network was constructed by Cytoscape (version 3.4.0) (Shannon et al., 2003).

### Metabolic pathway and function enrichment analysis

The metabolic pathway analysis was performed *via* the webtool MetaboAnalyst 5.0 (Pang et al., 2021). The R package ClusterProfiler (version 4.2.1) (Wu et al., 2021) was used for gene function enrichment analysis. Each *p* value was corrected by the Benjamini-Hochberg method.

### Molecular docking

The protein structures of ALOX12 (ID: 3d3l), IKKB (ID: 3brt) and TRPV1 (ID: 3sui) were downloaded from RCSB PDB. The ligands and solvent molecules in protein structures were removed *via* AutoDockTools (v 1.5.6). 7,8-DHF (ID: 1880) were download from https://pubchem.ncbi.nlm.nih.gov. The corresponding metabolite structures of chrysin (ID: MOL002560) and DHC (ID: MOL008698) were download from TCSMP. AutoGrid4 in Autodock Vina (v 1.2.2) was used for molecular docking of the proteins (as receptors) and metabolites (as ligands). Default input parameters were used in all computations. Binding energy between each ligand and receptor was calculated by Autodock Vina.

### Statistical analysis

All values were expressed as mean ± SEM, and statistical analyses were implemented using GraphPad Prism 8. A one-way ANOVA followed by Tukey’s test was conducted to analyze the data for significant differences. *N* represents the number of mice in each group and the number of independent experiments. *p* < 0.05 indicated statistically significant.

## Results

### *Pinelliae rhizoma* exhibited anti-inflammatory efficacy in lipopolysaccharide-induced acute lung injury

**FIGURE 2:**
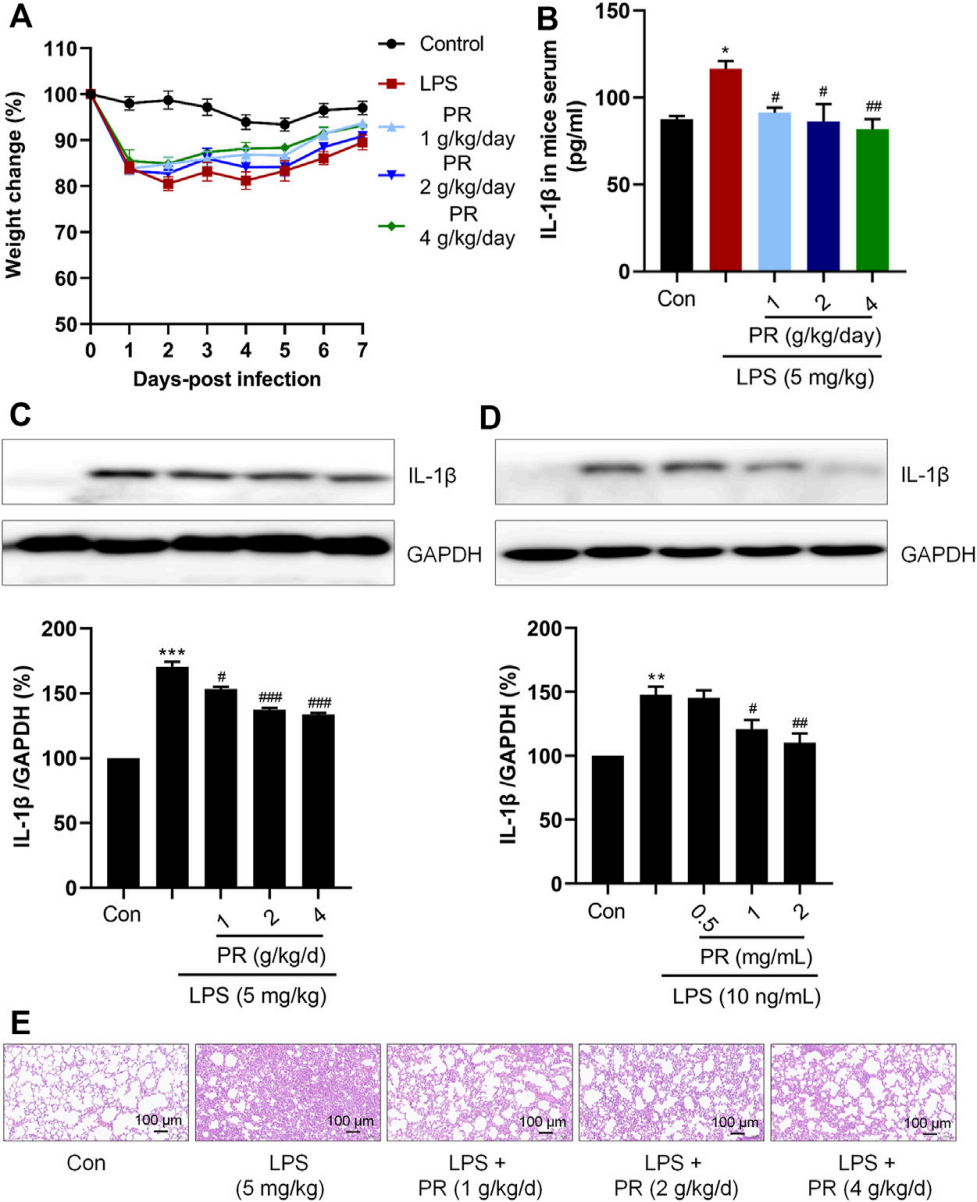
PR exhibits anti-inflammatory efficacy in LPS stimulation. **(A)** body weight change of mice in 7 days during administration. **(B)** Serum IL-1β levels were detected by Elisa kits (*n* = 5–6). **(C)** Immunoblot was employed to evaluate the expression of IL-1β in lung tissue of mice. **(D)** RAW264.7 cells were treated with LPS or/and PR for 12 h, followed by Western blot analysis using IL-1β antibody (*n* = 3). **(E)** Representative HE staining of lung tissue from ALI mice that were treated or untreated with PR (*n* = 6). Scale bar = 100 μm *p < 0.05, **p < 0.01, ***p < 0.001, ****p < 0.0001 vs. Control groups*; #p <* 0.05*, ##p <* 0.01*, ###p <* 0.001*, ####p <* 0.0001 vs. LPS-treated groups.

LPS-induced ALI mice model was treated with PR for 7 days. The weight changes of the mice were recorded daily, and the results showed that LPS reduced the body weight of mice, and PR treatment alleviated the weight loss in ALI mice and recovered the body weight in the following 7 days gradually (Figure 2A). Pro-inflammatory cytokine IL-1β is known to mediate the initiation of the immune response, which promotes the release of other pro-inflammatory cytokines and disrupts immune homeostasis (Kandasamy et al., 2019). Therefore, suppressing excessive IL-1β will help to attenuate the inflammation response. IL-1β is a secretory protein released by macrophages, so the effects of PR on the level of IL-1β were examined in serum and lung tissue in ALI mice. As expected, PR not only effectively reduced the concentration of IL-1β in serum (Figure 2B), but it also inhibited the expression of IL-1β in lung tissue (Figure 2C).

In this study, the dose of LPS was selected to be 10 ng/ml, and the dose of PR was in the range of 0.5 mg/ml to 2 mg/ml, according to the references and preliminary experiment (Zhang et al., 2015; Arikawa et al., 2016). Then, we detected the expression of IL-1β in RAW264.7 cells after treatment with LPS and PR. As shown in Figure 2D, co-treatment with PR markedly reversed LPS-increased expression of IL-1β *in vitro* and *in vivo*, suggesting that PR exerts an anti-inflammatory effect by acting on the upstream mediators of IL-1β in response to LPS stimulation. Besides, histopathological examination of lung tissue was conducted by H&E staining. Researchers observed that severe infiltration of inflammatory cells and neutrophils in the alveolar space, diffuse edema in alveolar spaces and interstitium, and the alveolar walls were congested and thickened in LPS-induced ALI. The PR treatment group showed clearly ameliorated lesions in lung tissue with increased alveolar air space and reduced infiltration of inflammatory cells (Figure 2E).

### *Pinelliae rhizoma* inhibited the activation of endoplasmic reticulum stress and NLRP3 inflammasome

**FIGURE 3:**
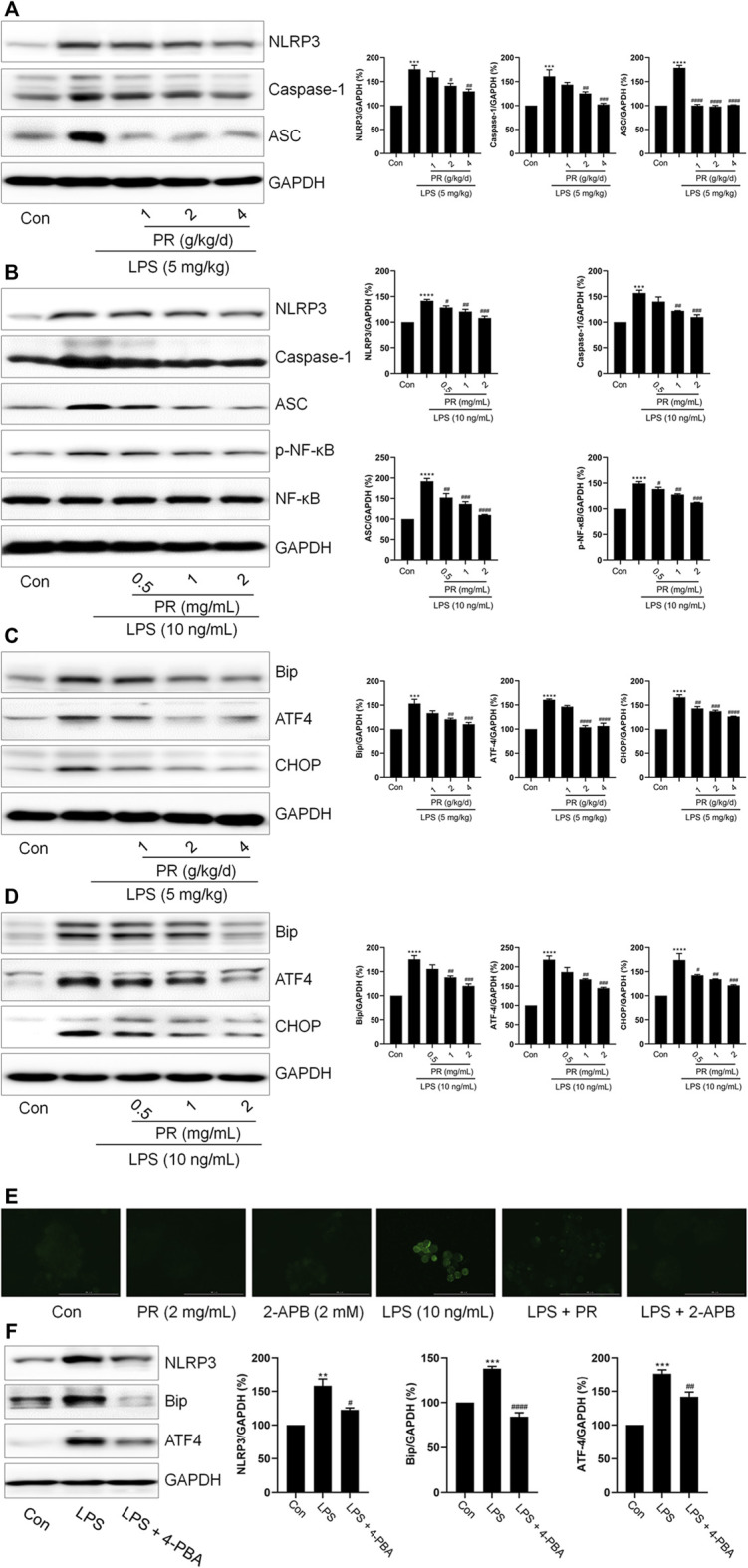
PR inhibits LPS-induced ER stress and NLRP3 inflammasome activation. **(A)** The expressions of IL-1β, NLRP3, Caspase-1, and ASC in the lung tissue of mice were determined by Western blot (*n* = 3). **(B)** RAW264.7 cells were challenged with LPS and treated with PR for 12 h. The expressions of IL-1β, NLRP3, Caspase-1, and ASC in RAW264.7 cells were examined by Western blot. (*n* = 3). **(C)** The expression levels of ATF4, Bip, and CHOP in lung tissue were detected by Western blot. **(D)** RAW264.7 cells were challenged with LPS and treated with PR for 12 h. The expressions of ATF4, Bip, and CHOP in RAW264.7 cells were measured by Western blot (*n* = 3). **(E)** Intracellular Ca^2+^ content was detected with Fluo 4-AM. Scale bar = 100 μm. **(F)** After 12 h LPS treatment with/without 4-PBA co-treatment, the expressions of Bip, ATF4, and NLRP3 were detected by Western blot (*n* = 3). *p < 0.05, **p < 0.01, ***p < 0.001, ****p < 0.0001 vs. Control groups*; #p <* 0.05*, ##p <* 0.01*, ###p <* 0.001*, ####p <* 0.0001 vs. LPS-treated groups.

Given that ER stress and NLRP3 are critical in LPS-induced ALI, we investigated relevant protein expressions *via* immunoblotting. As shown in Figures 3A,B, LPS stimulation elevates NLRP3, Caspase-1, and ASC levels in RAW264.7 cells and lung tissue. Next, interestingly, PR co-treatment decreased the levels of NLRP3, Caspase-1, and ASC in LPS-treated RAW264.7 cells and lung tissue (Figures 3A,B). Meanwhile, another significant inflammation-related signaling pathway known as the NF-κB was stimulated by LPS, while PR treatment efficiently reduced its phosphorylation (Figure 3B). Also, co-treatment with PR significantly reversed LPS-increased the expressions of ATF4, CHOP, and Bip (Figures 3C,D). Further, collectively, these data demonstrated that PR demonstrates a significant inhibitory effect on ER stress and NLRP3 activation *in vitro* and *in vivo*. In addition, RAW264.7 cells were stained with Fluo-4 AM to detect changes in intracellular Ca^2+^ levels after LPS and PR treatments. Our results showed that the cytoplasmic Ca^2+^ loading in the LPS-treated group was significantly enhanced, while the cytoplasmic Ca^2+^ content in the PR and 2-APB (the inhibitor of calcium ion) treated groups decreased (Figure 3E). To investigate whether ER stress regulates NLRP3 inflammasome activation, we used 4-PBA (the inhibitor of ER stress) to inhibit ER stress and examined the expression of ER stress and NLRP3-related proteins. Our data showed that 4-PBA inhibited the expression of Bip and ATF4, while down-regulating the expression of NLRP3 (Figure 3F). These results displayed that inhibition of ER stress suppressed NLRP3 inflammasome activation, that is, NLRP3 activation is closely related to ER stress.

### Effects of *Pinelliae rhizoma* on serum metabolites of lipopolysaccharide-induced acute lung injury model

**FIGURE 4:**
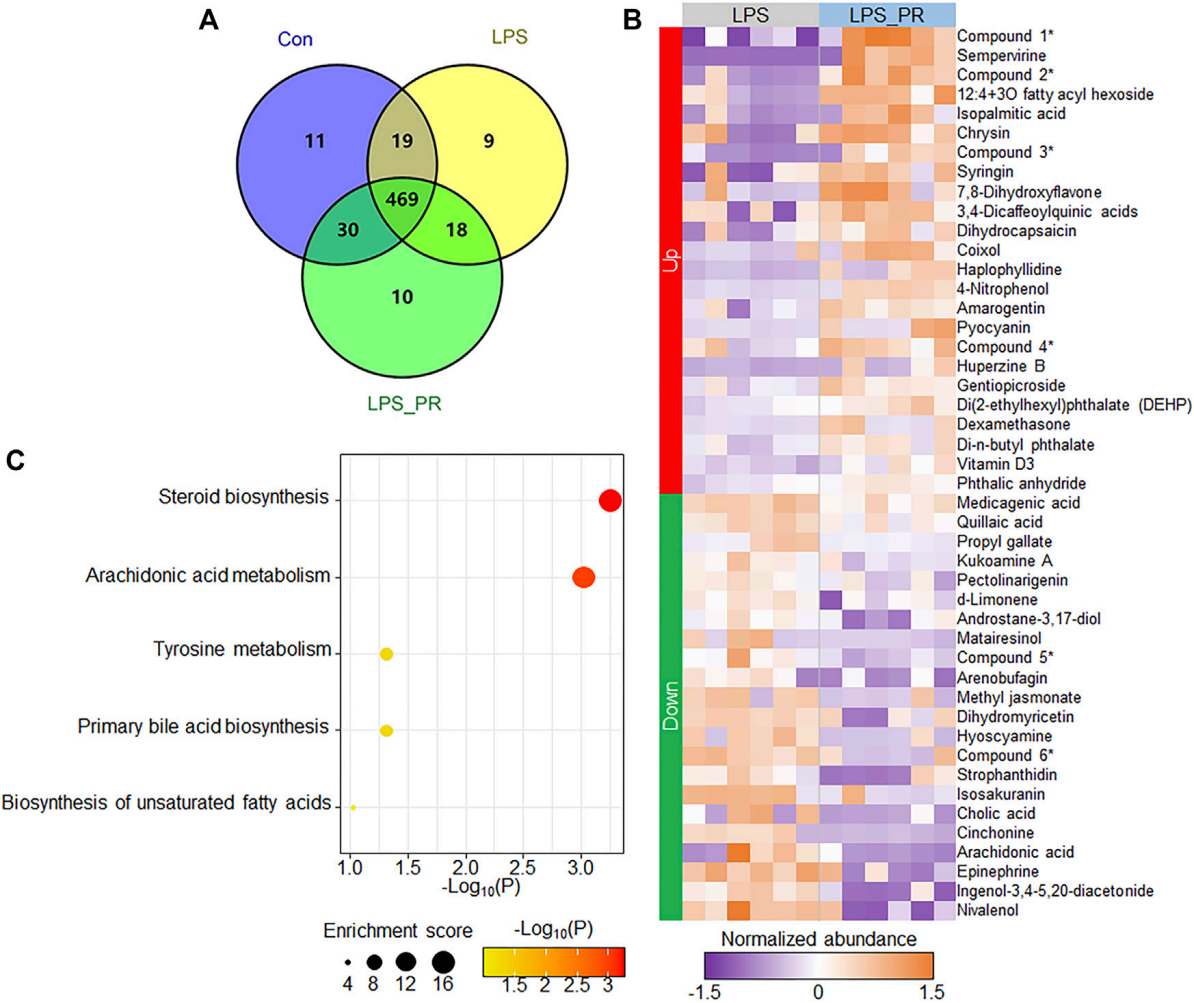
PR changed the serum metabolites of LPS-stimulated mouse. **(A)** Number of identified metabolites in the three types of experimental samples. **(B)** Heatmap of 46 differentially expressed metabolites in LPS and LPS_PR. Purple represents low expression and orange represents high expression. Due to the long names of some ingredients, abbreviations are used for display (indicated by asterisks on the right side of the heat map). Compound 1: [(1S,6S,7R)-6-acetyloxy-1-(3-methylbutanoyloxy)spiro[4a,5,6,7a-tetrahydro-1H-cyclopenta[c]pyran-7,2′-oxirane]-4-yl]methyl 3-methylbutanoate; Compound 2: Pyrrolo[1,2-a]pyrazine-1,4-dione, hexahydro-3-(1-methylethyl)-; Compound 3: (1S,2R,4aS,6aR,6bR,10S,12aR,14bS)-1,2,6b,9,9,12a-hexamethyl-10-[(2R,3R,4S,5S,6R)-3,4,5-trihydroxy-6-methyloxan-2-yl]oxy-2,3,4,5,6,6a,7,8,8a,10,11,12,13,14b-tetradecahydro-1H-picene-4a,6a-dicarboxylic acid; Compound 4: (2S,3S,4S,5R,6S)-3,4,5-trihydroxy-6-(5-hydroxy-4-oxo-2-phenylchromen-7-yl)oxyoxane-2-carboxylic acid; Compound 5: 2,4,6-trihydroxy-5-[1-(4-hydroxy-1,1,4,7-tetramethyl-1a,2,3,4a,5,6,7a,7b-octahydrocyclopropa[h]azulen-7-yl)-3-methylbutyl]benzene-1,3-dicarbaldehyde; Compound 6: (1S,4aR,6aS,6bR,10R,11R,12aR,14bS)-1,10,11-trihydroxy-2,2,6a,6b,9,9,12a-heptamethyl-1,3,4,5,6,6a,7,8,8a,10,11,12,13,14b-tetradecahydropicene-4a-carboxylic acid. **(C)** Pathway enrichment results of differentially expressed metabolites. The darker the color, the more significant the *p* value.

Serum metabolome is a new approach that combined TCM theory with advanced systematic pharmacology technology to study the TCM. In order to consider whether some bioactive ingredients were found in PR, which play the anti-inflammatory roles in the regulation of ER stress and NLRP3 inflammasome, serum metabolome can be used to overcome the difficulty of multi-ingredients and multi-targets in the research of PR. After quality control and data processing, the PLS-DA result showed that samples in the same group were clustered together, and the three groups were clearly distinguished (Supplementary Figure S1A), indicating that high-confidence metabolomes were obtained which could be used for downstream analysis. A total of 98,067 peaks were detected in the three types of experimental samples, 566 metabolites were identified (Figure 4A and Supplementary Table S1), and most (82.9%, 469/566) metabolites were co-identified in three groups. Next, subsequently, we focused on the expression changes of metabolites in the LPS model before and after PR administration. Significant differences were found between the LPS and LPS_PR groups (Supplementary Figure S1B). The differential analysis identified a total of 46 differentially expressed metabolites (*p* < 0.05 and VIP >1), of which 24 were up-regulated and 22 were down-regulated (Figure 4B and Supplementary Table S1), suggesting that PR alters mouse serum metabolome in the presence of ALI. These differentially expressed metabolites tend to be involved in steroid biosynthesis and arachidonic acid metabolism pathways (Figure 4C), which are closely related to inflammation (Riad et al., 2002; Mariotto et al., 2007; Wang et al., 2019).

### Network pharmacology revealed important bioactive ingredients in *Pinelliae rhizoma* and their possible targets

**FIGURE 5:**
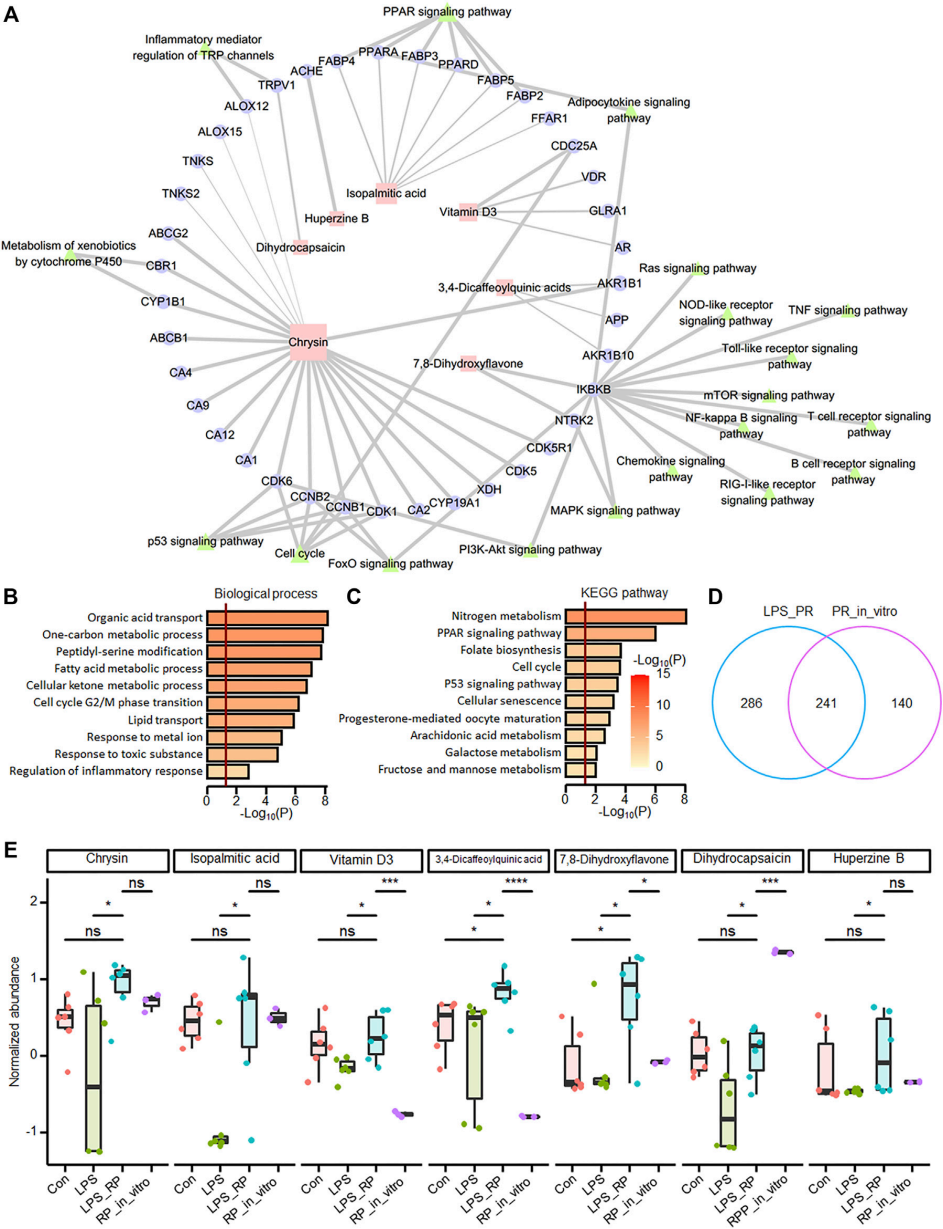
Network pharmacological analysis showed that potential anti-inflammatory ingredients are found in PR. **(A)** PR network pharmacology. 39 targets of seven metabolites and KEGG pathways closely related to inflammation were displayed in the network. Squares represent metabolites, circles represent potential targets, and triangles represent KEGG pathways. The size of the shape is positively correlated with the degree, and the thickness of the line is positively correlated with probability. **(B)** Functional enrichment results of targets of differentially expressed metabolites. **(C)** Pathway enrichment results of targets of differentially expressed metabolites. The darker the color, the more significant the *p* value. The red vertical bar indicates that *p* value is 0.05. **(D)** overlap of identified metabolites in LPS_PR and PR *in vitro*. **(E)** Abundance of seven metabolites in four groups in Con, LPS, LPS_PR and PR *in vitro*. Welch’s *t*-test was performed for compare the expression differences of metabolites between each two groups. *p < 0.05, **p < 0.01, ***p < 0.001, ****p < 0.0001.

In order to explore the PR regulatory network, through network pharmacological analysis (*see* methods), we found that seven of the 24 up-regulated metabolites in LPS_PR regulate a total of 39 ALI-related targets, of which chrysin, isopalmitic acid, and vitamin D3 demonstrate the most targets, 22, 7, and 4, respectively (Figure 5A and Supplementary Table S2). Functional enrichment analysis showed that these 39 targets tend to be involved in the functions closely related to lipid and energy metabolism and transport, cell cycle, response to stimulation and regulation of inflammatory response (Figure 5B), and tend to participate in metabolic pathway, PPAR signaling pathway, cell cycle, and p53 signaling pathway (Figure 5C). To explore which of these seven metabolites (Supplementary Data Sheet S2) are ingredients in PR, we identified all ingredients in PR using UHPLC-QE MS. 381 ingredients are found in PR, including 97 terpenoids, 65 alkaloids, 38 flavonoids, 26 phenylpropanoids, 23 phenols, 21 amino acid derivatives, and 111 other components (Supplementary Table S3). 241 metabolites were identified in both LPS_PR and PR_*in_vitro* (Figure 5D). Among them, chrysin, isopalmitic acid, DHC, and 7,8-DHF demonstrate relatively high expression levels in PR *in vitro* (Figure 5E), suggesting that these ingredients in PR may regulate and treat inflammation through the above corresponding pathways. Since 7,8-DHF and chrysin demonstrate very similar chemical results, we speculated that the high abundance of 7,8-DHF in LPS_PR may be derived from the metabolism of chrysin in PR. These results suggested that these ingredients in PR may regulate and treat inflammation through the above corresponding pathways.

**FIGURE 6:**
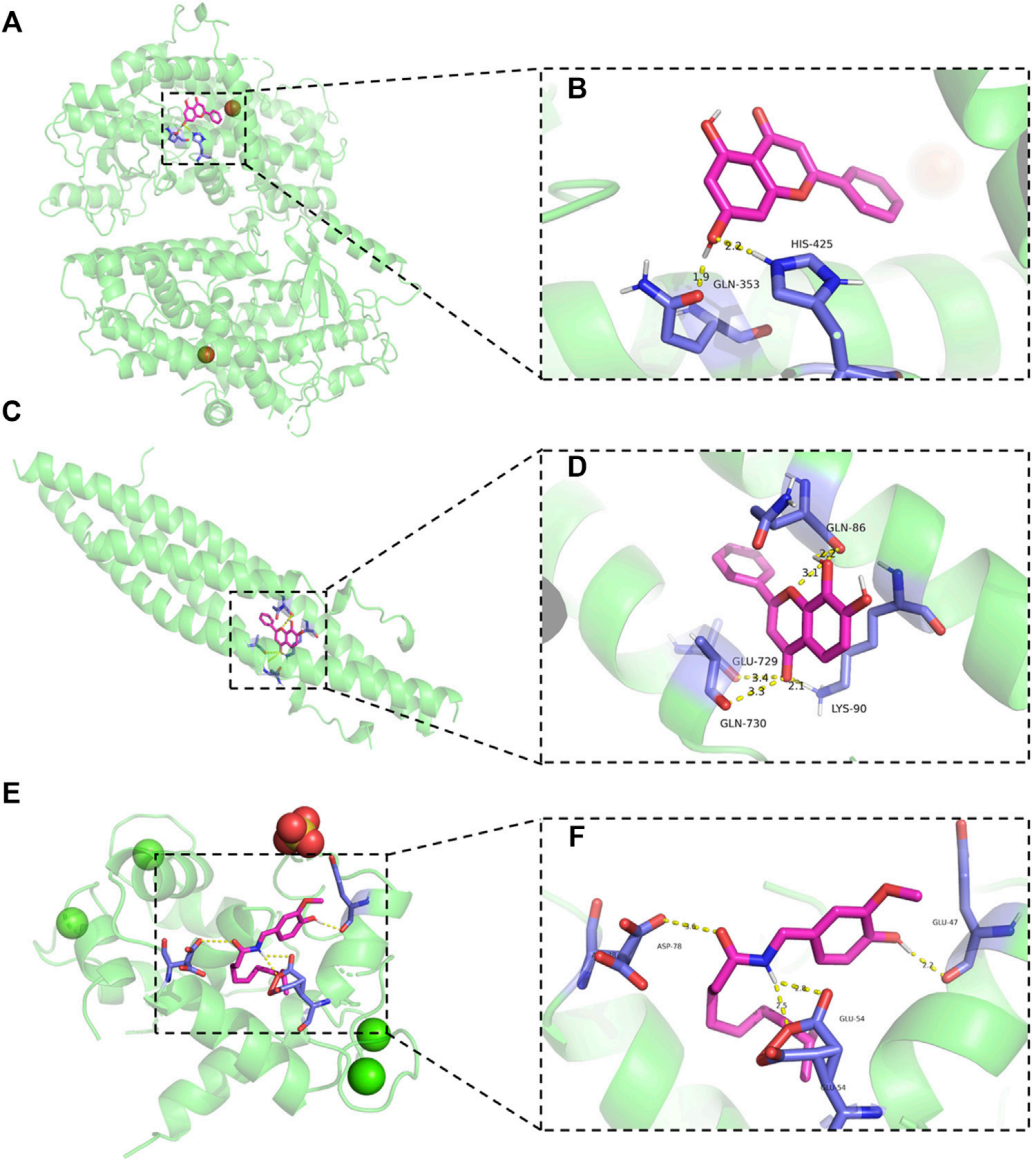
Molecular docking results. **(A**,**B)** Schematic diagram of the binding mode of chrysin to ALOX12 protein (left panels) and details of the binding pose (right panels). **(C**,**D)** Schematic diagram of the binding mode of 7,8-DHF to IKKB protein (left panels) and details of the binding pose (right panels). **(E**,**F)** Schematic diagram of the binding mode of DHC to TRPV1 protein (left panels) and details of the binding pose (right panels).

To further explore whether genes closely related to inflammation could be bound by ingredients and potential metabolic outcomes from PR, we carried out molecular docking on chrysin with ALOX12, 7,8-DHF with IKKB, and DHC with TRPV1. Binding energies between chrysin and ALOX12, 7,8-DHF with IKKB, and DHC with TRPV1 are −6.45, −6.37, and −5.91 kcal/mol, respectively. Chrysin binds to ALOX12 in the pocket between the one chain (Figure 6A). Hydroxyl groups of chrysin form hydrogen bonds with GLN-353 and HIS9425 in ALOX12 (Figure 6B). 7,8-DHF binds to IKKB in the pocket between the two chains (Figure 6C). Carbonyl group of 7,8-DHF form hydrogen bonds with GLU-729 and GLN-730 in chain A (Figure 6D). Hydroxyl groups of 7,8-DHF form hydrogen bonds with GLN-86, and carbonyl group of 7,8-DHF form hydrogen bonds with LYS-90 in chain B (Figure 6D). DHC binds to TRPV1 in the pocket between the two chains (Figure 6E). Hydroxyl group of DHC form hydrogen bonds with GLU-47, and amino groups of DHC form hydrogen bonds with GLU-54 in chain A (Figure 6F). Carbonyl group of DHC form hydrogen bonds with ASP-78 in chain B (Figure 6F).

### Bioactive ingredients of *Pinelliae rhizoma* suppressed endoplasmic reticulum stress and NLRP3 inflammasome

**FIGURE 7:**
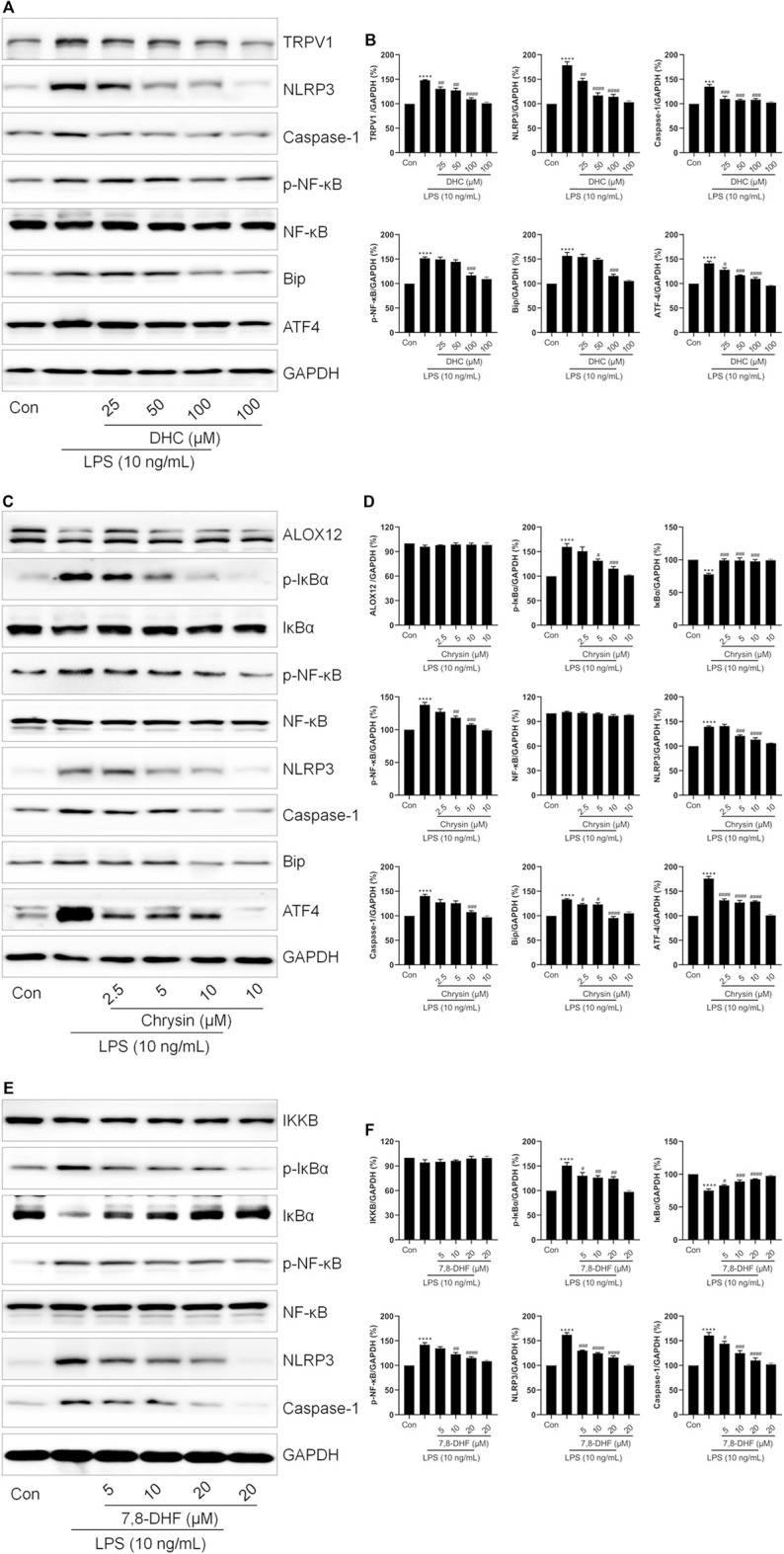
Validation of the effects of DHC, chrysin, and 7,8-DHF on inflammation-related proteins. RAW264.7 cells were exposed to LPS and treated with DHC, chrysin, or 7,8-DHF for 12 h. **(A**,**B)** The effects of DHC on the expressions of TRPV1, NLRP3, Caspase-1, p-NF-κB, Bip, and ATF4 were examined by Western blot (*n* = 3). **(C**,**D)** The effects of chrysin on the expressions of ALOX12, p-IκBα, IκBα, p-NF-κB, NLRP3, Caspase-1, Bip, and ATF4 were detected by Western blot. (*n* = 3). **(E**,**F)** Western blot was employed to detect the effects of 7,8-DHF on the expressions of IKKB, p-IκBα, IκBα, p-NF-κB, NLRP3 and Caspase-1 (*n* = 3). *p < 0.05, **p < 0.01, ***p < 0.001, ****p < 0.0001 vs. Control groups*; #p <* 0.05*, ##p <* 0.01*, ###p <* 0.001*, ####p <* 0.0001 vs. LPS-treated groups.

Next, we continued to investigate the effects of chrysin, DHC and 7,8-DHF on ER stress, respectively. LPS induced ER stress and NLRP3 inflammasome activation in cells, while both DHC (Figures 7A,B) and chrysin (Figures 7C,D) significantly down-regulated the expression of NLRP3, Caspase-1, Bip and ATF4. Also, co-treatment with DHC significantly reversed LPS-increased the expressions of TRPV1 and p-NF-κB (Figures 7A,B). As shown in Figures 7C,D, compared with the LPS group, the expressions of p-IκBα and p-NF-κB were distinctly decreased, but that of IκBα was obviously increased in the chrysin-treated group. Also, no changes were found in ALOX12 expression between the Control, LPS-treated, and the chrysin-treated group (Figures 7C,D). Moreover, compared with the LPS group, the expressions of p-IκBα, p-NF-κB, NLRP3, and Caspase-1 were significantly decreased, and the expression of IκBα was increased in the 7,8-DHF-treated group (Figures 7E,F). Also, no changes were found in IKKB expression among the LPS-treated group and the 7,8-DHF treated group (Figures 7E,F). Taken together, these results indicated that chrysin, DHC, and 7,8-DHF are the effective ingredients of PR regulating inflammation-related signaling pathways and ion channels.

## Discussion

ALI is one of the most common critical diseases in clinics, with rapid onset and high mortality (He et al., 2021). Severe ALI or improper treatment could lead to further ARDS. Previous studies showed that TCM demonstrates its unique advantage in the treatment of lung injury, which is mainly reflected in the regulation of immune function (Ding et al., 2020). PR has been used to treat lung diseases for thousands of years, and it effectively suppressed pro-inflammatory cytokines and relieved airway inflammation (Du et al., 2016; Hu et al., 2019). This study identified that LPS triggered ER stress and increased cytoplasmic Ca^2+^ content *via* activating Bip/ATF4/CHOP signaling pathway. Also, PR effectively relieves ALI induced by LPS, mainly *via* suppressing ER stress-mediated NLRP3 inflammasome and excessive expression of IL-1β. In addition, the bioactive ingredients of PR exerting anti-inflammatory effects were screened. Among them, chrysin, DHC, and 7,8-DHF significantly inhibited LPS-induced ER stress and NLRP3 inflammasome activation.

The infiltration of inflammatory cells and excessive release of pro-inflammatory cytokines are key events that trigger ALI (Belchamber and Donnelly, 2017). With the pathogenic microorganisms invading the lungs, cytokines were released to repair lung injury; however, overwhelming cytokines are destructive and cause serious injury (Parekh et al., 2011). Therefore, inhibition of cytokine release is necessary to alleviate lung injury. Consistent with previous reports and our hypothesis, this study confirmed that PR alleviated LPS-induced lung pathological injury and inhibited the excessive expression of cytokine IL-1β. Besides, NLRP3 mediates the outbursts of pro-inflammatory cytokines and PR down-regulated the expression of NLRP3 in lung tissue and RAW264.7 cells, indicating that PR limited the activation of NLRP3 inflammasome caused by LPS. Also, importantly, ER stress-mediated NLRP3 inflammasome also participates in cytokine release (Talty et al., 2019). The ER is a reservoir of Ca^2+^, and a large amount of Ca^2+^ flow from the ER into mitochondria and cytoplasm during ER stress, resulting in mitochondrial Ca^2+^ overload and damage (Liu et al., 2018). Next, subsequently, damaged mitochondria produce excess mtROS and mtDNA and cause cardiolipin damage, thus promoting the assembly and activation of the NLRP3 inflammasome to release cytokines (Lee et al., 2012). In addition, elevated Ca^2+^ levels of cytoplasmic also directly activate the NLRP3 inflammasome (Murakami et al., 2012). Hence, inhibition of the activity of ER stress-mediated NLRP3 inflammasome is a fresh target for the cure of ALI. Not surprisingly, PR significantly down-regulated Bip/ATF4/CHOP signaling pathways in LPS-treated cells and lung tissue, and it reduced cytoplasmic Ca^2+^ loading in LPS-stimulated cells. Together, these results proved the effect of PR in abolishing inflammation mainly through inhibiting ER stress and suppressing the NLRP3 inflammasome.

Bioactive ingredients of TCM are presented in the form of prototype components or metabolites in the body (Shi et al., 2016). To observe whether bioactive ingredients in PR were absorbed by mice and entered the blood to exert potent anti-inflammatory effects after administration, we determined the metabolites in mouse serum before and after LPS stimulation, and the ingredients of PR. 46 differentially expressed metabolites were identified, which tend to participate in inflammation-related pathways, such as steroid biosynthesis and arachidonic acid metabolism pathways. Network pharmacology and molecular docking analysis screened three prototype and metabolic components of PR and their target proteins, chrysin and ALOX12, DHC and TRPV1, 7,8-DHF, and IKKB. Further *in vitro* experiments demonstrated that chrysin, DHC, and 7,8-DHF treatment suppress ER stress and the activation of NLRP3 inflammasome.

Chrysin, 7,8-DHF, and DHC demonstrate significant biological properties, including anti-inflammatory and immune modulation (Chen et al., 2011; Janyou et al., 2017; Byun et al., 2021). The latest research shows that chrysin improved LPS-induced ALI in mice by inhibiting ER stress and NLRP3 inflammasome activation (Chen et al., 2021). Meanwhile, 7,8-DHF can inhibit the LPS-induced release of inflammatory mediators in RAW264.7 cells (Park et al., 2012). TRPV1 is a non-selective cation channel (Zhai et al., 2020). The activation of TRPV1 promotes Ca^2+^ influx, leads to intracellular calcium overload, and affects ER stress and a series of inflammatory responses (Stock et al., 2012; Stueber et al., 2017). From this, it seems that the suppression of ER stress by PR may benefit from the inhibition of TRPV1 activation by DHC. Next, interestingly, chrysin and 7,8-DHF demonstrated no effect on the expression of their target proteins ALOX12 and IKKB but inhibited the NF-κB signaling pathway. However, ALOX12 accelerates inflammatory responses and promotes cytokine production through the p38 mitogen-activated protein kinase (MAPK) and NF-κB pathways (Funk and Cyrus, 2001; Nieves and Moreno, 2008). Also, accordingly, PR inhibition of NF-κB phosphorylation in RAW264.7 cells may be related to chrysin and 7,8-DHF.

Further, collectively, chrysin, DHC, and 7,8-DHF are the potential key bioactive ingredients of PR to regulate inflammatory response by inhibiting ER stress-mediated NLRP3 inflammasome activation, thereby conferring protection to the lungs and maintaining lung function and lung homeostasis. Given that NLRP3 is one of the targets for the treatment of COVID-19 and inhibition of NLRP3 inflammasome activation could effectively alleviate infection-induced lung injury (Xian et al., 2021), this study not only elucidates the mechanism and components of PR in the treatment of ALI, but it will also contribute to the development of COVID-19 therapeutic drugs.

## Conclusions

**FIGURE 8:**
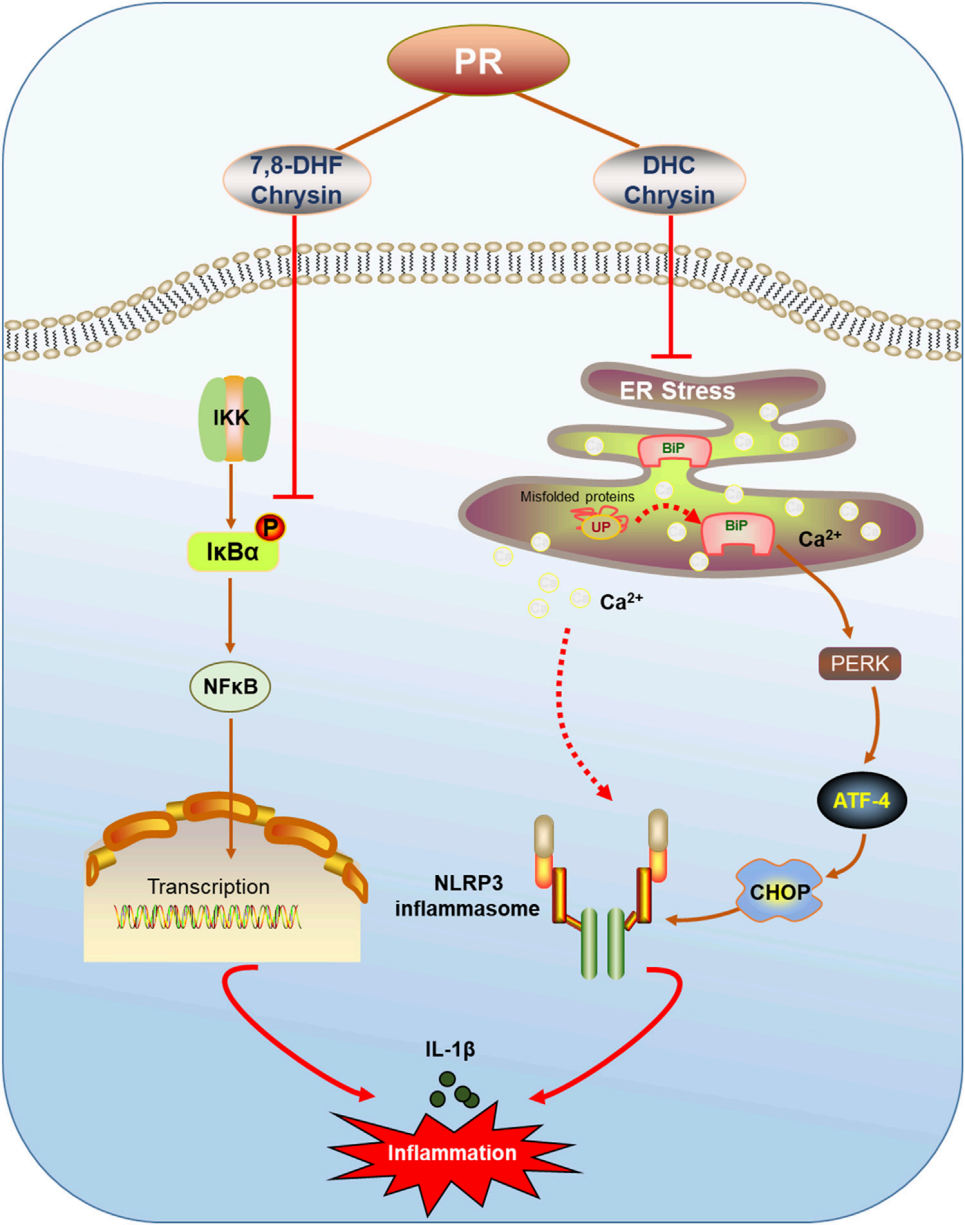
Schematic of PR relieving ALI by inhibiting ER stress. PR and its active ingredients alleviated inflammatory injury *via* regulating ER stress-mediated NLRP3 and NF-κB signaling pathways.

In summary, this study confirmed that LPS infection indeed caused acute inflammatory damage in mouse lung, and it is accompanied with the enhancement of IL-1β contents and the activation of the NLRP3 inflammasome in lung tissue and macrophagocyte, all of which are remarkably ameliorated by PR treatment. PR not only obviously reversed Bip/ATF4/CHOP-mediated ER stress, but it also significantly attenuated LPS-induced activation of the NLRP3 inflammasome. Based on metabolome analysis and molecular docking, chrysin, 7,8-DHF, and DHC were found to notably suppress LPS-induced ER stress and NLRP3 inflammasome activation (Figure 8). Hence, this research provided a theoretical basis for the clinical application of PR to treat ALI, and these bioactive ingredients of PR would be promising therapeutic drugs for the treatment of ALI.

## Data Availability

The original contributions presented in the study are included in the article/Supplementary Material, and further inquiries can be directed to the corresponding authors.

## Abbreviations

2-APB, 2-aminoethyl diphenylborinate; 4-PBA, sodium 4-phenylbutyrate; 7,8-DHF, 7,8-dihydroxyflavone; ALI, acute lung injury; ARDS, acute respiratory distress syndrome; ATF4, activating transcription factor (ATF)-4; Ca^2+^, calcium ions; CHOP, C/EBP homologous protein; COVID-19, coronavirus disease 2019; DHC, dihydrocapsaicin; ER, endoplasmic reticulum; GRP78, glucoseregulated protein 78; IL-1β, interleukin-1β; IREα, inositol-requiring enzyme 1α; LPS, lipopolysaccharide; NF-κB, nuclear factor kappa-B; NLRP3, nucleotide-binding domain, leucine-rich repeat-containing receptor, pyrin domain-containing 3; PERK, protein kinase (PKR)-like ER kinase; PR, *Pinelliae rhizoma*; SARS-CoV-2, severe acute respiratory syndrome coronavirus 2; TCM, traditional Chinese medicine; UPR, unfolded protein response.
