# Supplementary figures and images for: Pinelliae rhizoma alleviated acute lung injury induced by lipopolysaccharide via suppressing endoplasmic reticulum stress-mediated NLRP3 inflammasome
Source: Front Pharmacol. 2022 Aug 15;13:883865. doi: 10.3389/fphar.2022.883865 (PMC9421150; doi:10.3389/fphar.2022.883865)

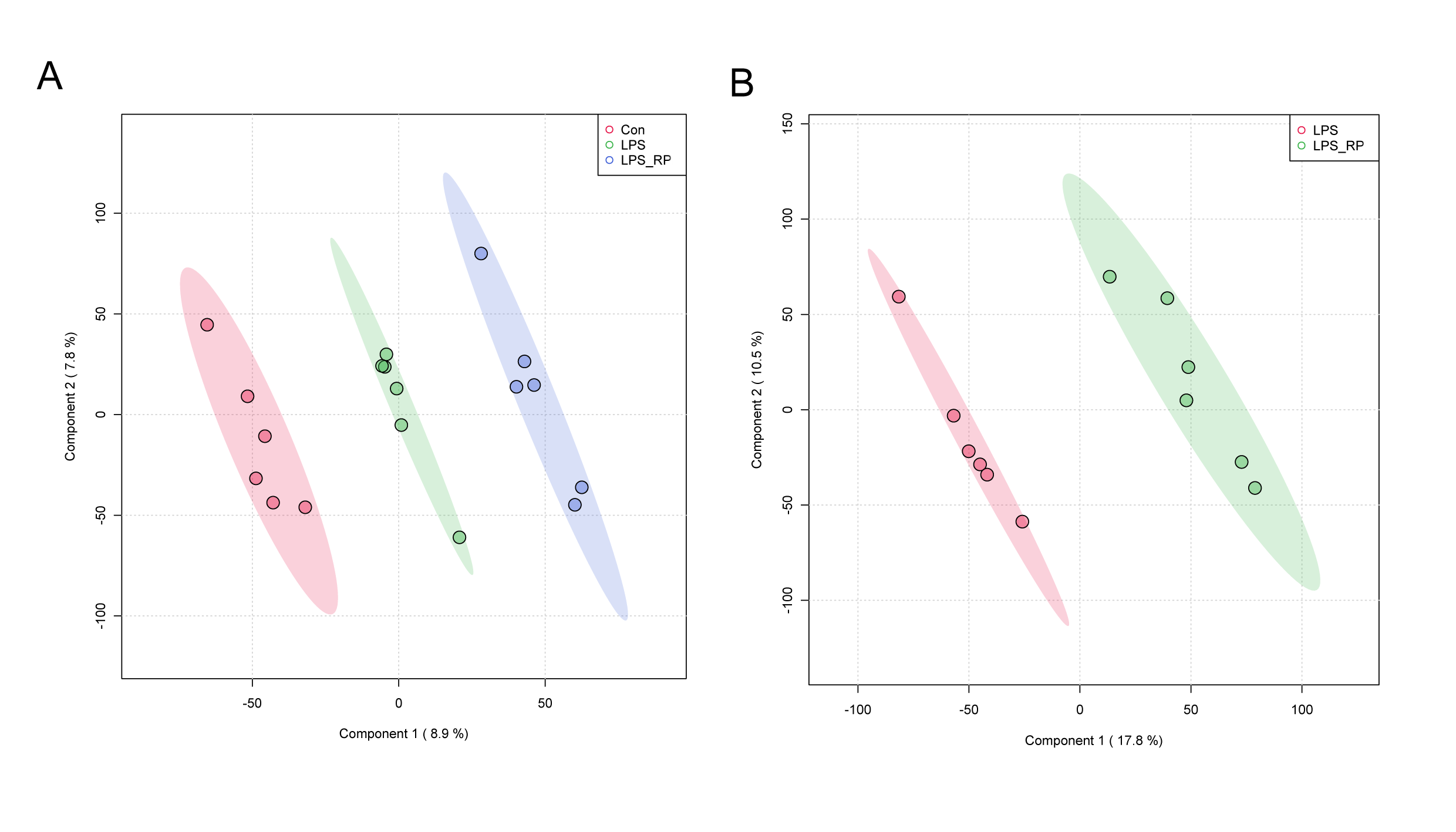

Supplement: Supplementary file 3 [file Image1.TIF]
