# Supplementary material for: Pinelliae rhizoma alleviated acute lung injury induced by lipopolysaccharide via suppressing endoplasmic reticulum stress-mediated NLRP3 inflammasome
Source: Front Pharmacol. 2022 Aug 15;13:883865. doi: 10.3389/fphar.2022.883865 (PMC9421150; doi:10.3389/fphar.2022.883865)

**Supplementary materials 2**


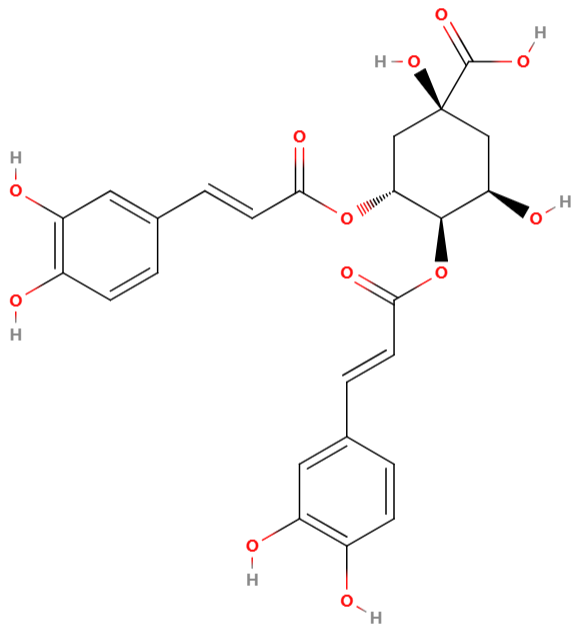

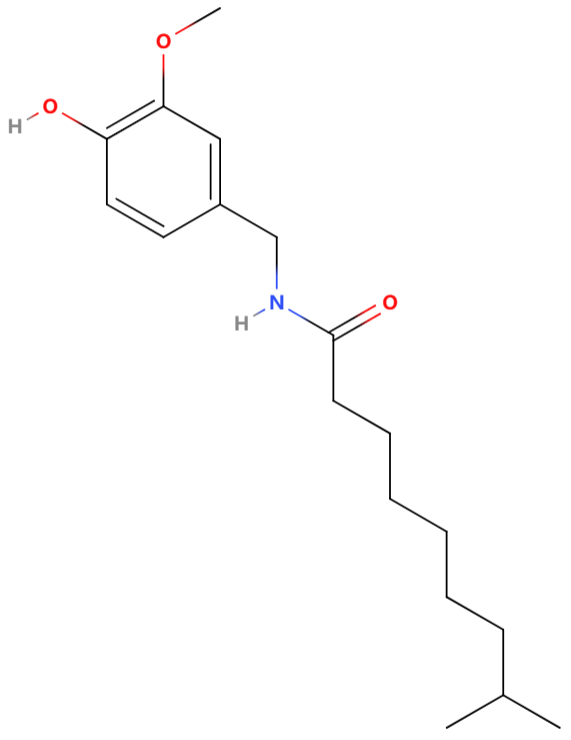

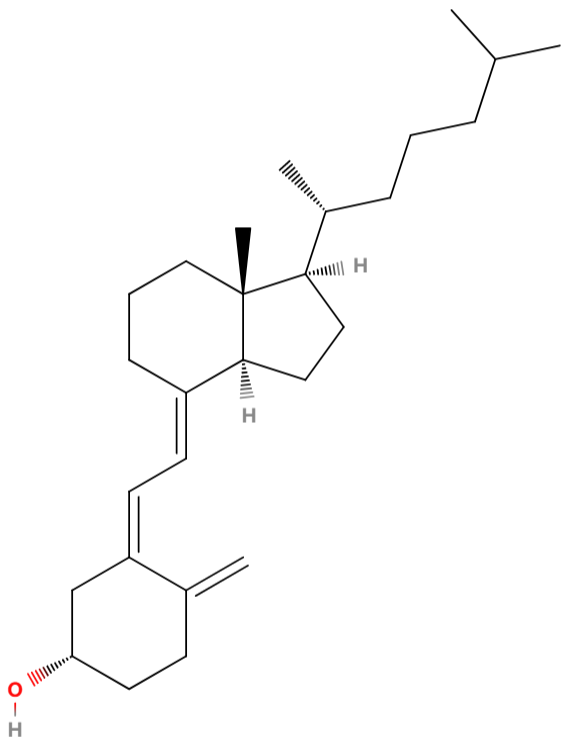


Huperzine B


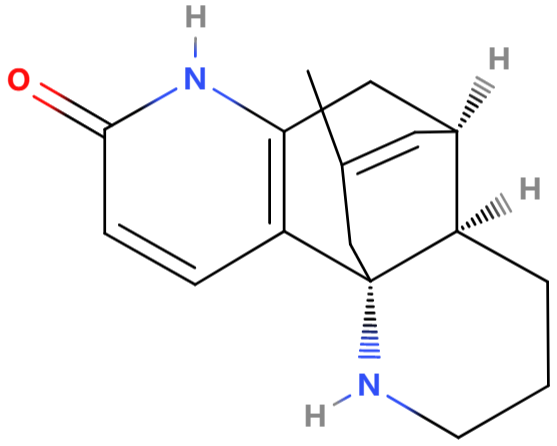


7,8-Dihydroxyflavone


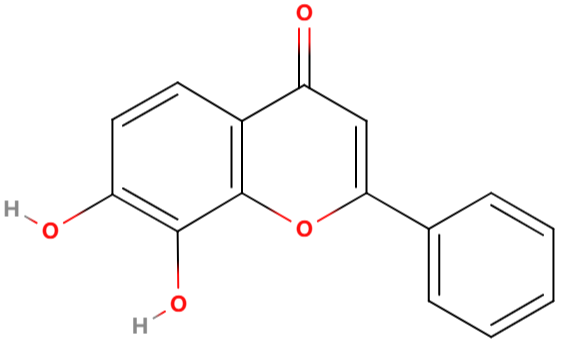


Vitamin D3

Dihydrocapsaicin

3,4-Dicaffeoylquinic acid

Isopalmitic acid


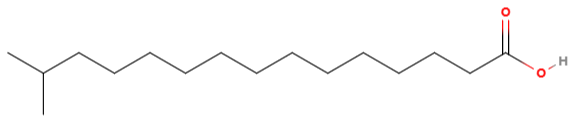


Huperzine B


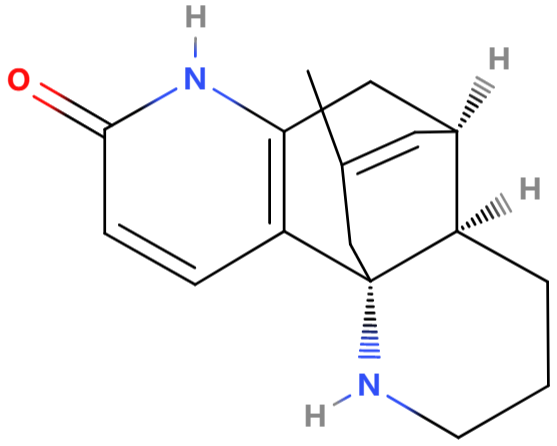


Chrysin


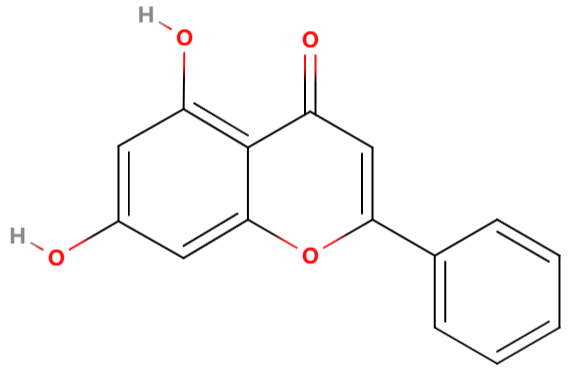

Supplement: Supplementary file 5 [file DataSheet2.docx]
