# Supplementary material for: Pinelliae rhizoma alleviated acute lung injury induced by lipopolysaccharide via suppressing endoplasmic reticulum stress-mediated NLRP3 inflammasome
Source: Front Pharmacol. 2022 Aug 15;13:883865. doi: 10.3389/fphar.2022.883865 (PMC9421150; doi:10.3389/fphar.2022.883865)

Supplementary materials 1


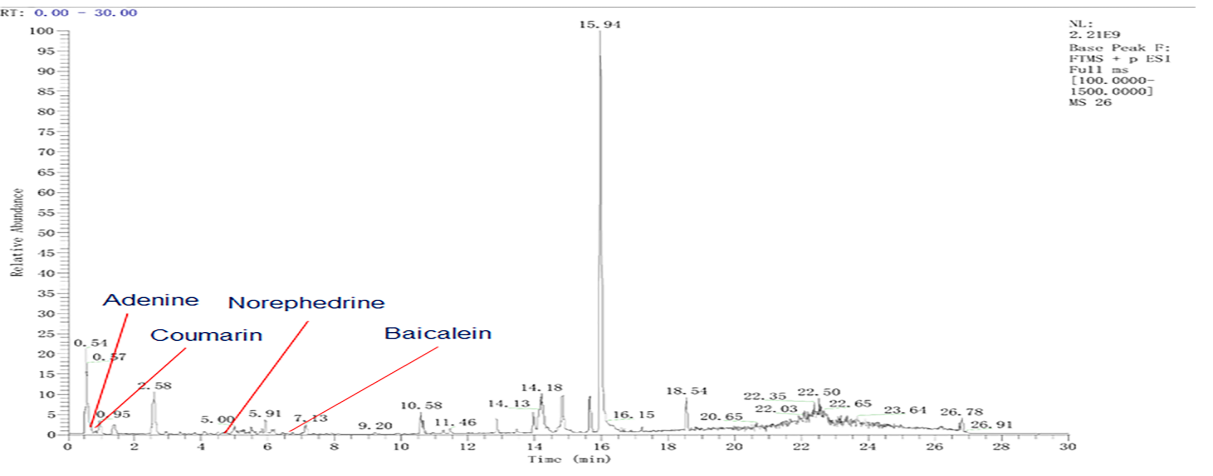


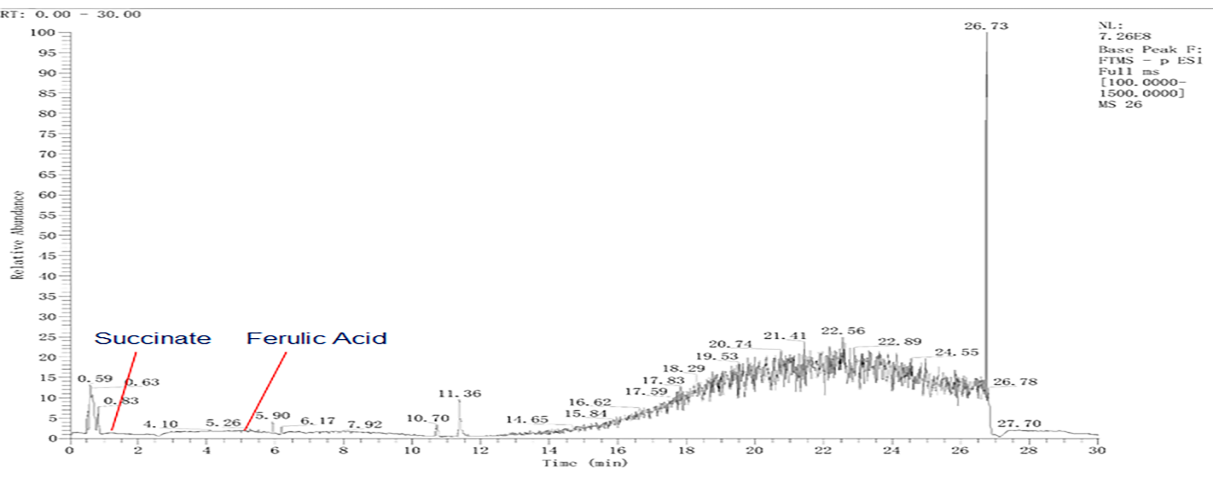


**Figure 1** Base peak ion (BPI) chromatograms of *Pinelliae Rhizoma* samples under different ion modes.

Table 1 Specific information on characteristic compounds of *Pinelliae Rhizoma.*


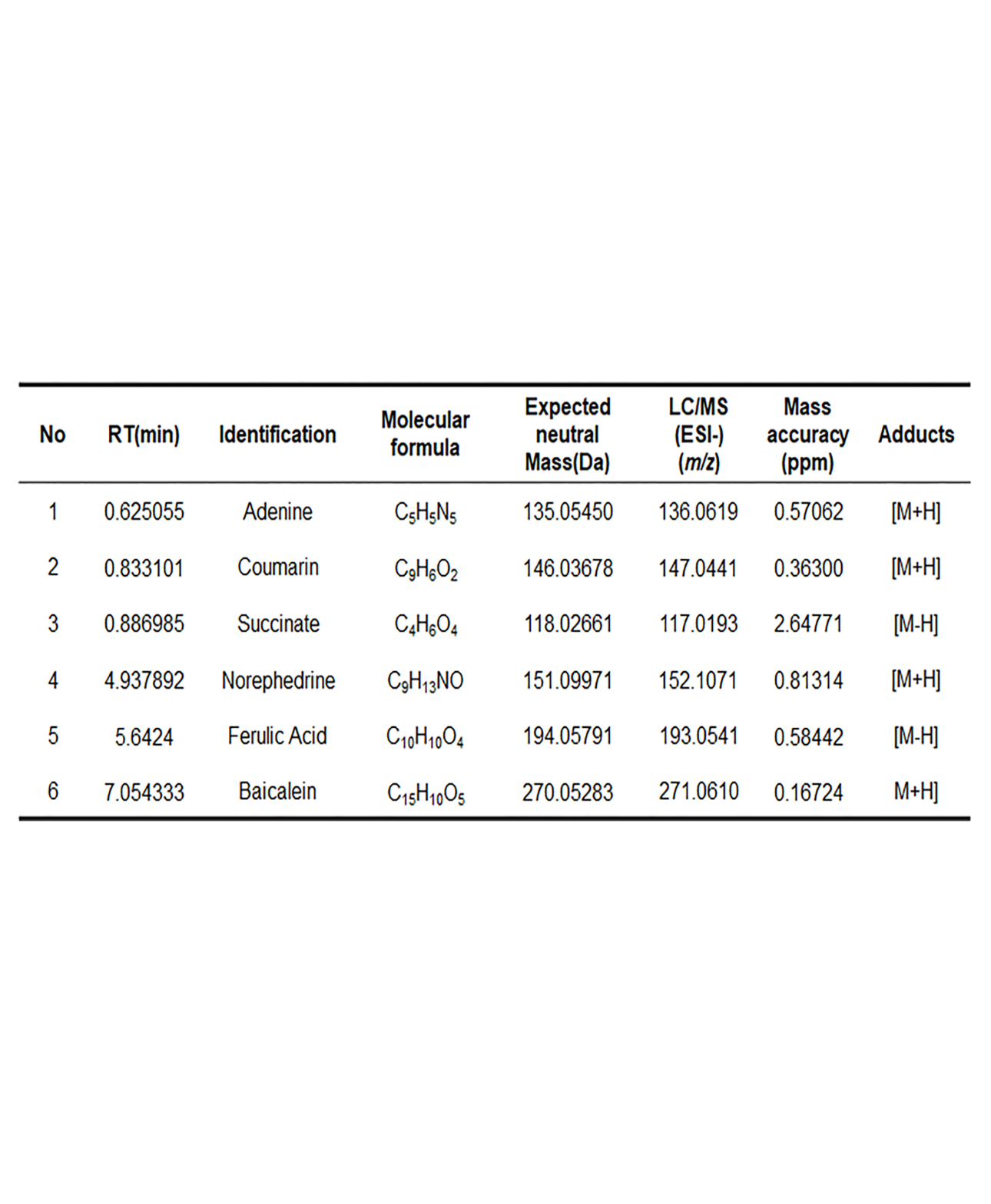


1-Adenine （C_5_H_5_N_5_）


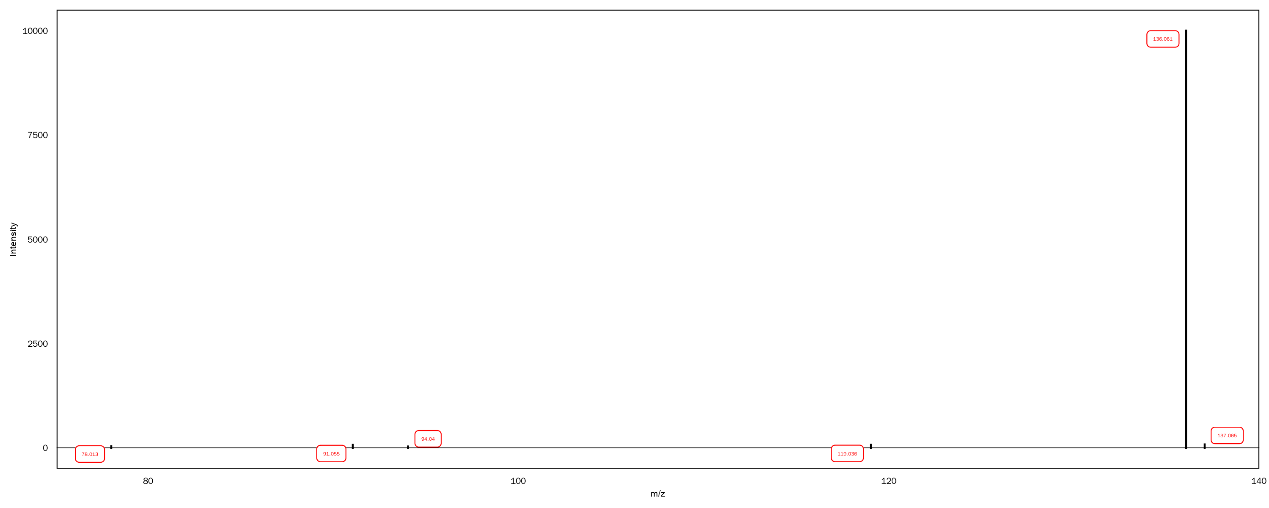


2- Coumarin （C_9_H_6_O_2_）


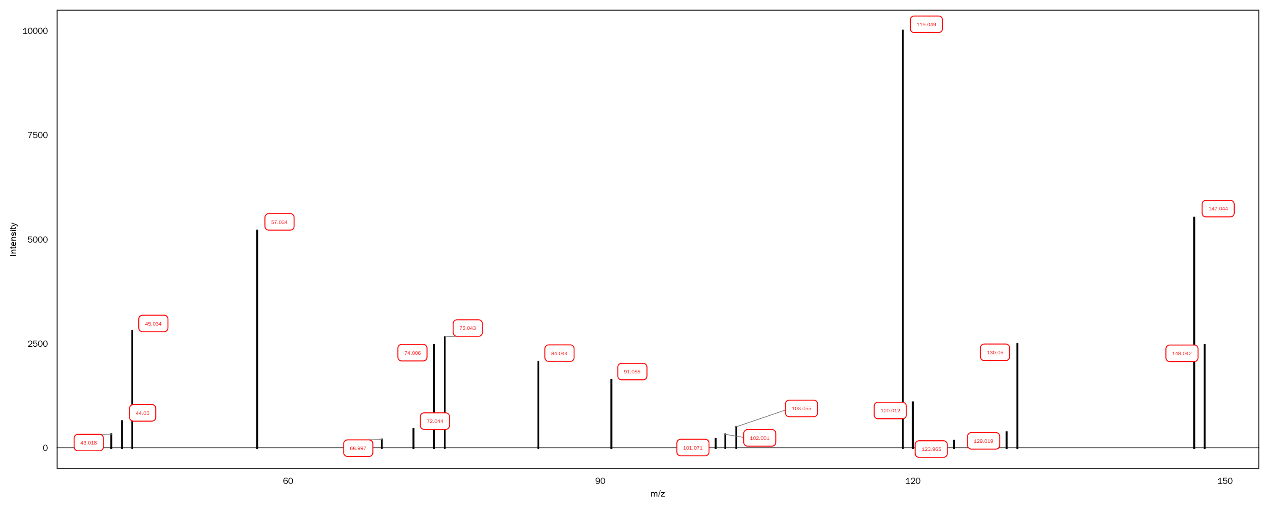


3- Succinate （C_4_H_6_O_4_）


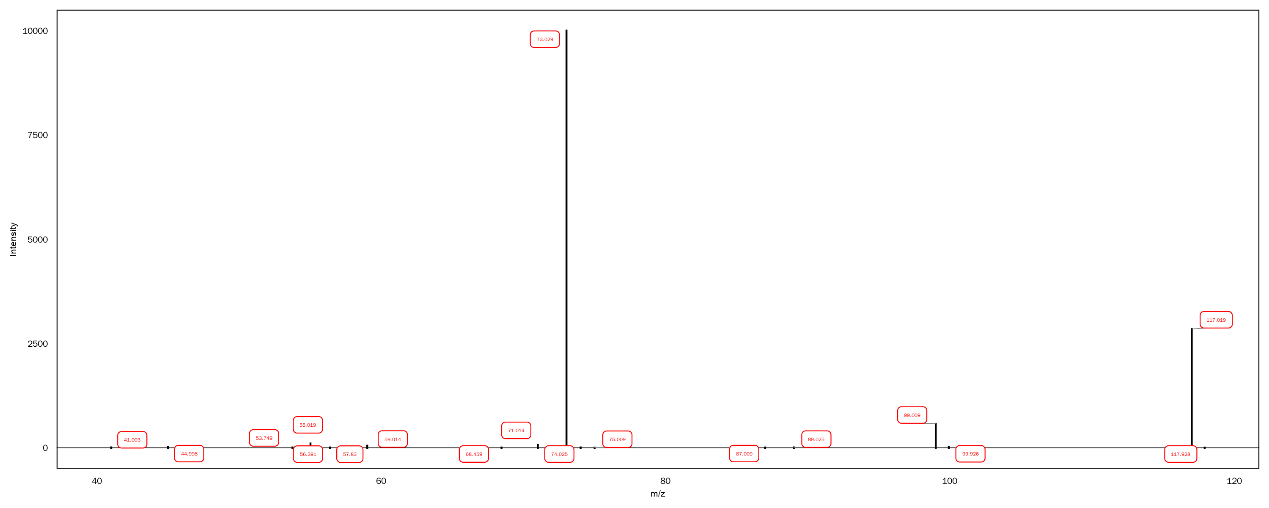


4- Norephedrine （C_9_H_13_NO）


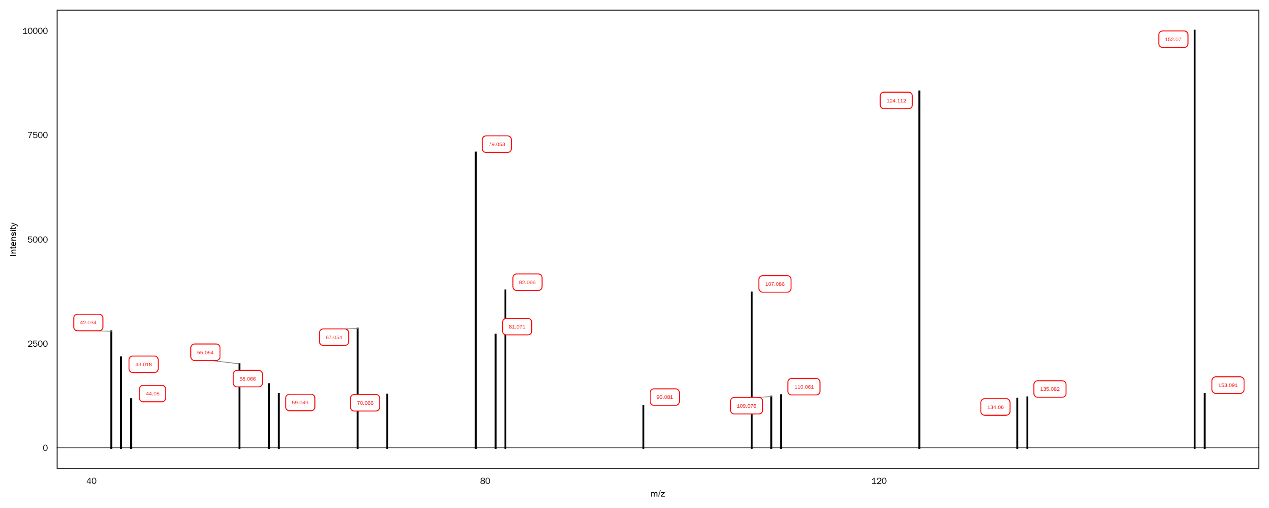


5- Ferulic Acid （C_10_H_10_O_4_）


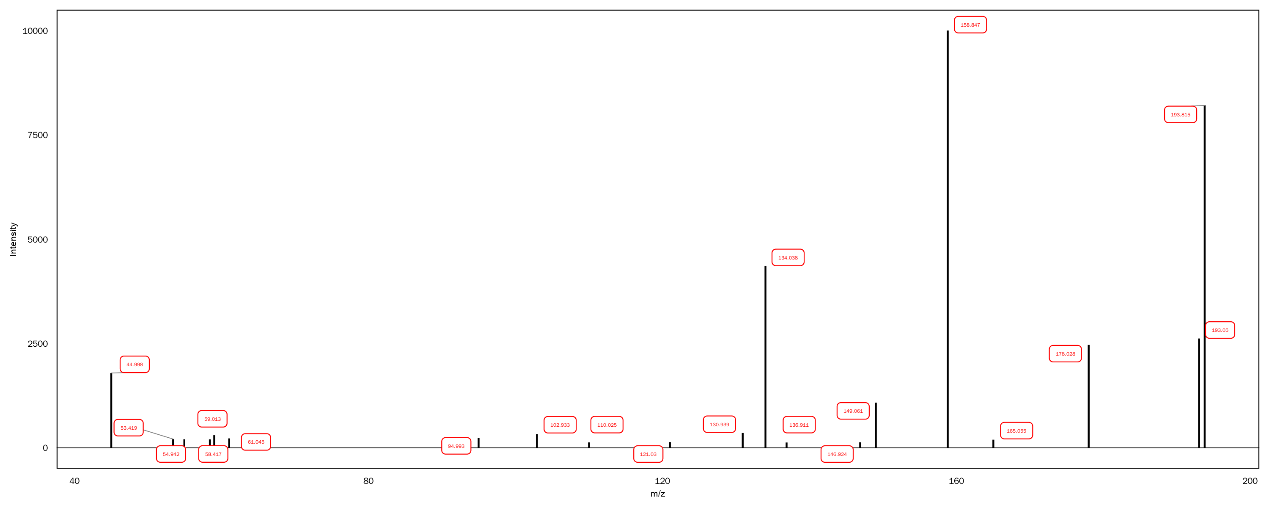


6- Baicalein （C_15_H_10_O_5_）


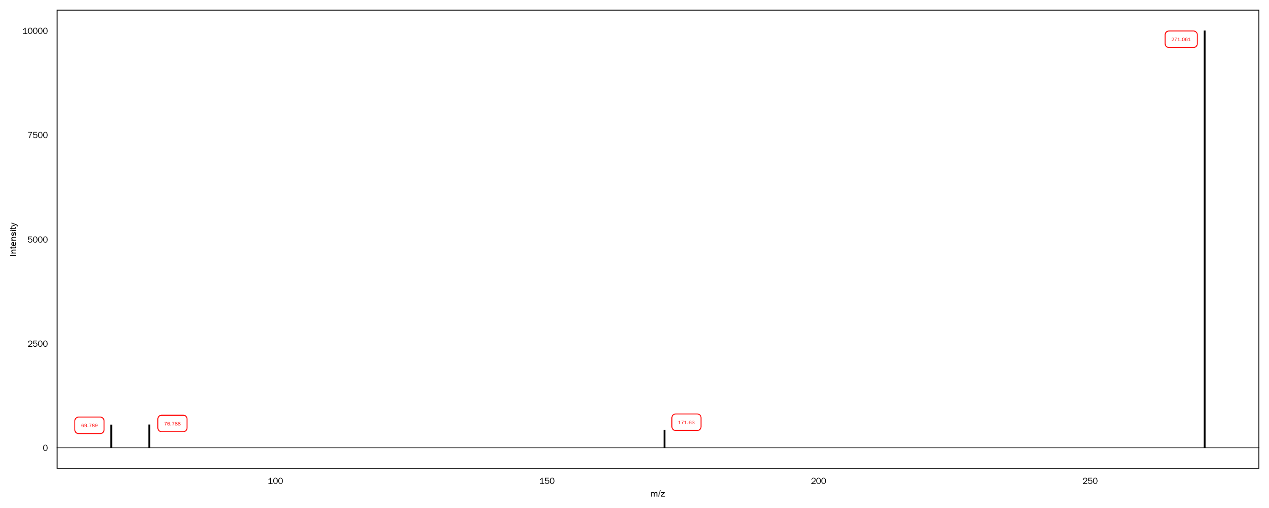

Supplement: Supplementary file 6 [file DataSheet1.docx]
